# Trauma and Depression among North Korean Refugees: The Mediating Effect of Negative Cognition

**DOI:** 10.3390/ijerph15040591

**Published:** 2018-03-25

**Authors:** Subin Park, Yeeun Lee, Jin Yong Jun

**Affiliations:** 1 Department of Research Planning, Mental Health Research Institute, National Center for Mental Health, Seoul 04933, Korea; subin-21@hanmail.net (S.P.); tasarang1010@korea.ac.kr (Y.L.); 2 Department of Social Psychiatry and Rehabilitation, National Center for Mental Health, Seoul 04933, Korea

**Keywords:** North Korean refugees, depression, early trauma, negative automatic thoughts, path analysis

## Abstract

North Korean refugees experience adaptation difficulties, along with a wide range of psychological problems. Accordingly, this study examined the associations between early traumatic experiences, negative automatic thoughts, and depression among young North Korean refugees living in South Korea. Specifically, we examined how different factors of negative automatic thoughts would mediate the relationship between early trauma and depressive symptoms. A total of 109 North Korean refugees aged 13–29 years were recruited from two alternative schools. Our path analysis indicated that early trauma was positively linked with thoughts of personal failure, physical threat, and hostility, but not with thoughts of social threat. The link with depressive symptoms was only significant for thoughts of personal failure. After removing all non-significant pathways, the model revealed that early traumatic experiences were positively associated with depressive symptoms (ß = 0.61, 95% CI = 0.48–0.73) via thoughts of personal failure (ß = 0.17, 95% CI = 0.08–0.28), as well as directly (ß = 0.44, 95% CI = 0.27–0.59). Interventions that target negative cognitions of personal failure may be helpful for North Korean refugees at risk of depression.

## 1. Introduction

Many North Korean refugees often report having experienced traumatic events in North Korea, especially during their escape from North Korea [1] and after settling in South Korea [2]. In particular, research has found that 49.3% of adult North Korean refugees have experienced or witnessed life-threatening events [3]. Among young North Korean refugees, 71% reported having experienced traumatic incidents in the past, such as the death or arrest of family members or hearing about these events, as well as being physically abused by family members or acquaintances [4]. As a result of having experienced such traumatic events, North Korean refugees represent a mentally vulnerable population. Indeed, these traumatic experiences have been consistently found to be associated with psychiatric problems and low life satisfaction among North Korean refugees. In general, the frequency and severity of traumatic experiences have been found to predict post-traumatic stress disorder, anxiety, and depression [4,5,6,7,8,9]. Indeed, depression is the most frequently reported mental health problem among adult North Korean refugees, with the prevalence of depression ranging from 29% [9] to 49% [10].

One possible mechanism through which traumatic experiences contribute to the affective symptoms of traumatized refugees may be the development and maintenance of negative cognitions. The cognitive theory of depression posits that repeated exposure to uncontrollable negative events that an individual fails to escape may alter how an individual perceives and interprets their own life events [11,12,13]. Specifically, when individuals experience a negative event, they attempt to understand and explain its causes. If negative events happen repeatedly and pervasively, they begin to interpret negative events as the consequences of their own actions and perceive them as unchangeable and generalizable to other domains (i.e., an internal, stable, and global attributional explanation) [13,14]. The frequently employed attributional explanations can negatively bias the victim’s general beliefs about themselves and about the world [14,15], from which automatic thoughts with negative themes arises, so-called “streams of consciousness cognitions” [16] (e.g., I will have an accident; I’ve failed my life). In particular, negative events that happened early in an individual’s life can crystallize more stable negative cognitions [14]. The negative cognition, in turn, acts as a cognitive vulnerability to the onset and persistence of affective disorders when facing prospective negative stressors [17,18], disenabling the person’s active coping with stressful situations with hopelessness [11,12]. 

Cognitive theorists have further proposed the cognitive content-specificity hypothesis, which proposes the distinctiveness of cognitive contents underlying different types of psychiatric problems [19]. For instance, depression can be linked more with thoughts of personal loss and failure in the past tense, while anxiety is more related to future-oriented danger themes such as physical, psychological, or social threats [19,20]. Empirical data also supported the idea that the different contents of automatic thoughts are uniquely associated with each affective symptom, such as with anxiety and depression [16,21,22]. In this vein, there have been efforts to organize the structure of cognitions according to similarity in themes, such as danger, loss, and failure [23,24]. In such efforts, Schneiering and Rapee [25] found four distinct cognitive factors among frequent negative automatic thoughts—cognitions of social threat, physical threat, personal failure, and hostility. The factor structure was found to be consistent across age and gender, supporting the stable latent structure of automatic thoughts. 

There is a paucity of research examining the pathway through which early traumatic experiences influence the mental health outcomes of North Korean refugees. North Korean refugees settling in South Korea are unique in that they share the same ethnicity, language, historical backgrounds, and physical features with the host people (i.e., South Koreans). Thus, they undertake very high risks, including the risk of forced repatriation, and a long flight process through third countries [26], with the expectation of entering a new society that they will seamlessly join. However, North Korean refugees are often faced with unexpected cultural gaps and social discrimination for their origin in North Korea [26]. These unexpected difficulties may reduce their ability to recover from early traumatization [27], presumably through reactivating negative cognitive contents developed throughout past trauma in early years. Identifying the cognitive mechanisms underlying psychological sequelae of the widespread adversities that North Korean refugees undergo would give insights into the core psychological factor to intervene to decrease the risk of developing adverse affective symptoms in this high-risk group. Therefore, this study analyzed a model linking early trauma with depressive symptoms via negative cognitions in young North Korean refugees. We specifically tested how different types of negative automatic thoughts according to the ideational contents would mediate the relationship.

## 2. Materials and Methods

### 2.1. Participants

Participants were recruited from two schools for North Korean refugees designed to prepare students for the national qualification examinations for middle- or high-school graduation. Two schools, both located in Seoul, South Korea, volunteered to participate in this study. Of the 114 students who attended these schools, five students declined to participate, resulting in a total of 109 students who were ultimately enrolled in the study. After obtaining approval from the school principal regarding the research protocol, the investigators visited each school, explained the purpose of the study, and obtained informed consent from the participants. This study was approved by the human subjects institutional review board at the National Center for Mental Health (no. 2015-17 and no. 116271-2017-22). 

### 2.2. Measures

The socio-demographic variables assessed were sex, age, and perceived family economic status. Participants’ perceived family social economic status (SES) was assessed using a five-point Likert scale (low = 1, low-middle = 2, middle = 3, high-middle= 4, and high = 5). 

The Korean version of the Early Trauma Inventory Self Report-Short Form (ETISR-SF) [28,29] was used to assess the four domains of general experiences of traumatic events (11 items), as well as physical (5 items), emotional (5 items), and sexual abuse (6 items). The ETISR-SF consists of 27 items asking the experience of certain traumatic events that occurred before the age of 18 [28,29]. The presence of each traumatic experience was scored 1 (yes) or 0 (no), with total scores ranging from 0 to 27 (Cronbach’s alpha = 0.86). To examine the link between variations in early trauma and depressive symptoms, we used early trauma sum scores by counting up the number of events that had ever occurred to the individual based on the scoring method created by the developers of this scale [28]. By comparing the validity of different methods for obtaining scores, Bremner et al. [28] concluded that the most parsimonious and easiest method is to count up the number of events that had ever occurred. 

The Korean version of the Children’s Automatic Thoughts Scale (CATS) [24,30] was used to assess a wide range of negative self-statements. The CATS consists of four cognitive content subscales, including physical threat (6 items; e.g., “I’m going to have an accident”; Cronbach’s alpha = 0.947), social threat (10 items; e.g., “I’m worried that I’m going to get teased”; Cronbach’s alpha = 0.828), personal failure (10 items; e.g., “I’ve made such a mess of my life”; Cronbach’s alpha = 0.933), and hostility (6 items; e.g., “If someone hurts me, I have the right to hurt them back”; Cronbach’s alpha = 0.915). Although the original version of the CATS contains 40 items, the Korean version only contains 32 items, as 8 items were excluded that showed low explanatory and discriminative power. Items are rated on a five-point Likert scale, with responses ranging from 0 (strongly disagree) to 4 (strongly agree), with higher scores reflecting a higher level of negative automatic thoughts [24,30].

The Korean version of the Center for Epidemiologic Studies-Depression Scale (CES-D) [31,32,33] was used to measure depression. The CES-D consists of 20 items rated on a four-point Likert scale, with responses ranging from 0 (rarely or never) to 3 (mostly or always), and total scores ranging from 0 to 60. A cutoff score of ≥21 is recommended in community settings to screen for individuals with depressive symptoms, and a cutoff score of ≥25 is recommended for a clinical diagnosis of depression in Korean populations [31,32]. This study used a cutoff score of ≥21 to classify participants with significant depressive symptoms.

### 2.3. Statistical Analysis

Descriptive statistics were first calculated for all socio-demographic and psychological variables for the entire sample. Correlation analysis was first conducted to test inter-relationships between psychological variables, including early trauma, depressive symptoms, and four factors of negative automatic thoughts. Path analysis was initially conducted on early trauma, depressive symptoms, and four factors of negative automatic thoughts (i.e., personal failure, physical threat, social threat, and hostility) to test which type of negative automatic thoughts could mediate the relationship between early traumatic experiences and depressive symptoms in North Korean refugees. Considering the significant effects on mental health of sex, age, and socioeconomic status noted in previous studies of North Korean refugees (for a review, see [26]), we adjusted for such sociodemographic variables as covariates in our model. The categorical variable (i.e., sex) was converted to a dummy variable in the model (i.e., female = 0, male = 1). To make our final model more parsimonious, the final path analysis was conducted after removing non-significant pathways and parameters that do not contribute to the outcome variable of interest (i.e., depressive symptoms) in a significant way [34]. All analyses were conducted using SPSS software Version 22.0 (SPSS Inc., Chicago, IL, USA) and AMOS Version 24.0 (SPSS Inc., Chicago, IL, USA). For all statistical analyses, *p* < 0.05 was considered significant.

## 3. Results

Of the 109 participants (68 females, 41 males; mean age = 19.52, SD = 3.28 years; 16 with low SES, 19 with low-middle SES, 49 with middle SES, 17 with high-middle SES, and 8 with high SES), 52 (47.7%) had significant depressive symptoms (CES-D ≥ 21). Table 1 presents the frequency of endorsement for different types of early traumatic events and each of the four domains. Among the respondents, 90.8% were endorsed for at least one early traumatic event and they reported, on average, 6.0 (SD 5.0) traumatic events. The most frequent early traumatic events included physical abuse (76.1%; M = 2.1, SD = 1.7), notably being slapped in the face (49.5%), punched or kicked (47.7%), hit with a thrown object (41.3%), and general traumatic events (76.1%; M = 2.1, SD = 2.1), notably, separation of parents (43.1%). 

Table 2 presents correlations between early trauma and psychological variables. All main variables were significantly correlated with each other, with the exception of the correlation between early trauma and thoughts of social threat (r = 0.139, *p* = 0.149). 

**Figure 1:**
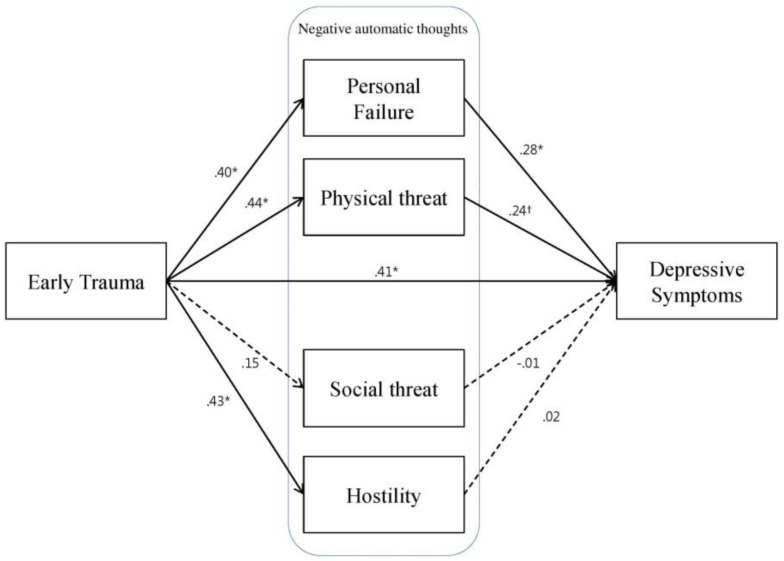
Path diagram illustrating the direct and indirect relationships between traumatic experiences, four types of negative automatic thoughts, and depressive symptoms, adjusting for sex, age, and family social economics status (Model 1). Standardized path coefficients (β) are reported in the model. Solid lines indicate significant associations and dotted lines indicate non-significant associations. ^†^ *p* < 0.10, * *p* < 0.05.

Our first model (Figure 1) revealed that early trauma was positively associated with depressive symptoms (ß = 0.63; 95% CI = 0.50–0.74; *p* = 0.01). The links between early trauma and negative automatic thoughts were all significant with the exception of thoughts of social threat (ß = 0.15; 95% CI = −0.05–0.32; *p* = 0.162). The association of depressive symptoms with negative automatic thoughts was significant only with those of personal failure (ß = 0.28; 95% CI = 0.08–0.48; *p* = 0.01), but not with those of physical threat (ß = 0.24; 95% CI = −0.04–0.47; *p* = 0.097), social threat (ß = −0.01; 95% CI = −0.13–0.15; *p* = 0.985), or hostility (ß = 0.02; 95% CI = −0.16–0.25; *p* = 0.971) at a *p* < 0.05 significance level. These results indicate that only thoughts of personal failure may be a potential mediator of the association between early trauma and depressive symptoms.

**Figure 2:**
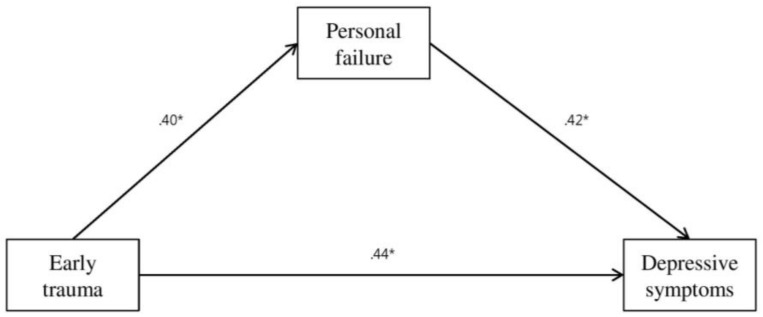
Path diagram illustrating the direct and indirect relationships between traumatic experiences, thoughts of personal failure, and depressive symptoms, adjusting for sex, age, and family social economics status (Model 2). Standardized path coefficients are reported in the model. Solid lines indicate significant associations. * *p* < 0.05. (as above mentioned)

Accordingly, our final path analysis (Figure 2) was conducted to test the mediating role of the thoughts of personal failure in the association between early trauma and depressive symptoms (Figure 2), with the thoughts of physical threat, social threat, and hostility removed from the initial model. The path analysis consistently showed that early trauma events were positively associated with depressive symptoms (ß = 0.61; 95% CI = 0.48–0.73; *p* = 0.01) via thoughts of personal failure (ß = 0.17; 95% CI = 0.08–0.28; *p* = 0.01) as well as directly (ß = 0.44; 95% CI = 0.27–0.59; *p* = 0.01). These results indicated partial mediation effects of cognition of personal failure. Fit indices indicated that the model had a moderately good fit to the data (GFI = 0.97, NFI = 0.91, CFI = 0.96, RMSEA = 0.09).

## 4. Discussion

The prevalence of significant depressive symptoms of North Korean refugees in our study (47.7%) was as high as that of previous reports that used the same CES-D cut-off score of 21 or above [10,35,36]. The results of the present study indicated that early traumatic experiences were positively associated with depressive symptoms in a sample of North Korean refugees, which is consistent with prior studies that have found an association between childhood trauma and depression in other community populations [37,38].

Consistent with prior findings on the high prevalence of traumatic experiences in refugee populations [39,40], and specifically among North Korean refugees (for a review, [26]), our findings showed a high frequency of early traumatic experiences among North Korean adolescents and young adults. In comparison with healthy South Korean adolescent and adult samples from previous studies [29,41], young North Korean refugees in this study had a generally higher number of early traumatic events that had ever occurred (5.99 vs. 2.33–3.65). Specifically, the average number of general traumatic events and experiences of sexual abuse they had experienced (2.13, 0.36 respectively) was more than twice higher than that of Korean adolescents and adults (0.86–0.96, 0.13–0.17, respectively) and the number of instances of physical abuse and emotional abuse they had ever experienced (2.11, 1.39, respectively) also tends to be higher than that in the Korean samples (0.80–1.79, 0.54–0.74, respectively). The relatively greater number of endorsements for different types of traumatic events in our young North Korean refugee sample suggests widespread traumatic experiences and childhood maltreatment during their early years.

Furthermore, this study extends the understanding of the close link between early traumatic events and later depressive symptoms in North Korea refugees [7,8,42] by elucidating its underlying cognitive mechanisms. As hypothesized, in general, negative automatic thoughts partially mediated the association between early trauma and depressive symptoms, regardless of participants’ sex, age, and socioeconomic status. The findings support cognitive theory of depression, which emphasizes the role of negative cognitions in linking past negative experiences, particularly in one’s early years, with affective symptoms [11,12,13]. Our findings are also consistent with empirical data that has shown robust association of childhood trauma caused both by family and non-family perpetrators with negative cognitive style [14], as well as with adult depression [38]. 

This study further demonstrates the differential mediating roles of negative cognitions depending upon their themes. More specifically, the links between early trauma of North Korean refugees and their depressive symptoms were partially mediated by thoughts of personal failure, but not by thoughts of physical and social threat or hostility. In general, the findings support cognitive specificity theory, which proposes that different themes of cognitions are uniquely linked with each psychopathology [19]. This is in line with the fundamental assumptions of cognitive theorists, which argue that personal failure can be more linked with depression than with anxiety or anger [19,20]. 

While being subjected to the accumulated adversities in North Korea or during their escape, North Korean refugees may develop the inclination to attribute negative events to one’s personal characteristics, such as ability, dispositions, or efforts, rather than to outside forces (i.e., internal attribution [13]), and this may cause them to engender the negative beliefs about the self and one’s failure. These negative cognitive contents can be reactivated, especially when the refugees are faced with unexpected stressors after resettlement in South Korea [17], including acculturative stress, social discrimination, and isolation [43]. The reactivated negative cognitions may create helplessness in the face of actively coping with new adversities and, consequently, may hinder successful psychosocial adaptations in the host country. This can, in turn, reaffirm negative cognitions of the self. Such a vicious cycle could be applied to other refugee groups, with exposure to a wide array of traumatic experiences in their home country and during their flight, in addition to acculturative stress in their host country [40]. 

Our analysis also showed that North Korean refugees’ early trauma was linked with activated thoughts of physical threat and hostility. Although these thoughts were not independently linked to depressive symptoms in our analysis, they may be linked to other psychological problems. Specifically, thoughts of physical threats can be linked with anxiety symptoms [19], whereas thoughts of hostility may be linked with anger and aggression by providing justification for such behaviors [44]. Future studies on the roles of the different contents of automatic thoughts in mediating early trauma and other psychiatric symptoms could extend the understanding of the cognitive mechanisms that underlie diverse psychological consequences of widespread trauma in refugee populations. 

These findings also extend prior studies that have examined mediators and moderators of the association between traumatic events and psychiatric symptoms. For example, Kim [45] found that post-traumatic stress disorder (PTSD) symptoms mediated the negative effect of past interpersonal traumatic events on other mental health comorbidities, such as depression and anxiety. In a moderator analysis, Park et al. [6] found that the relationship between the number of past traumas and PTSD symptoms was stronger for those with alexithymia. Our findings further highlight the mediating role of the negative cognitive processes in psychological sequelae of early trauma. It should be noted that maladaptive cognitive patterns characterized by negative views of the self, future, and the world predict the persistence of depression in later years (for a review, [17]). In this vein, our findings suggest that, in order to alleviate and prevent later depressive symptoms, it is necessary to develop interventions that target negative cognitions for North Korean refugees with early trauma. 

The present study has several limitations. First, given that this study was cross-sectional in nature, the temporal relationship among early traumatic experiences, negative cognition, and depressive symptoms could not be determined. It should be noted that mediation analyses are conditional on the validity of the path model being tested and cannot confirm causal model [46]. However, our model is a theoretically driven top-down model, which makes our mediation analysis more logically valid. Finally, given that schools volunteered to participate in this study, participants in the current study may not be representative of the larger population of young North Korean refugees.

## 5. Conclusions

Our data revealed that early traumatic experiences influence depressive symptoms, both directly and indirectly, via negative cognitions among young North Korean refugees. Given the high prevalence of depression as well as early trauma in this population, early depression screening is advisable for North Korean refugees with severe traumatic experiences. In particular, our findings suggest that interventions for North Korean refugees with a high susceptibility to depression should aim to correct negative cognitive processes, especially cognitions regarding personal failure.

## Figures and Tables

**Table 1:** Frequency of endorsement for the early trauma inventory item (N = 109).

Item	Frequency (%)
General trauma	76.1
T1. Natural disaster	10.1
T2. Serious accident	20.2
T3. Serious personal injury	27.5
T4. Serious injury/ illness of parent	22.0
T5. Separation of parents	43.1
T6. Serious illness/injury of sibling	10.1
T7. Serious injury of friend	14.7
T8. Witnessing violence	34.9
T9. Family mental illness	11.0
T10. Alcoholic parents	9.2
T11. Seeing someone murdered	10.1
Physical abuse	76.1
P1. Slapped in the face	49.5
P2. Burned with cigarette	34.9
P3. Punched or kicked	47.7
P4. Hit with thrown object	41.3
P5. Pushed or shoved	37.6
Emotional abuse	57.8
E1. Often put down or ridiculed	23.9
E2. Often ignored or made to feel you didn’t count	37.6
E3. Often told you are no good	20.2
E4. Most of the time treated in cold or uncaring way	30.3
E5. Parents fail to understand your needs	27.5
Sexual abuse	18.3
S1. Touched in intimate parts in way that was uncomfortable	13.8
S2. Someone rubbing genitals against you	4.6
S3. Forced to touch intimate parts	5.5
S4. Someone had genital sex against your will	6.4
S5. Forced to perform oral sex	2.8
S6. Forced to kiss someone in sexual way	2.8

**Table 2:** Correlations among main variables.

Variable	1	2	3	4	5	6
1. CES-D	1					
2. ETI total	0.60 **	1				
3. CATS-Personal failure	0.61 **	0.40 **	1			
4. CATS-Social threat	0.20 *	0.14	0.33 **	1		
5. CATS-Physical threat	0.61 **	0.45 **	0.72 **	0.21 *	1	
6. CATS-Hostility	0.53 **	0.43 **	0.67 **	0.22 *	0.75 **	1
Mean	21.3	6.0	7.1	11.3	6.3	3.6
Standard deviation	10.9	5.0	8.6	5.6	8.1	5.3

CES-D, Center for Epidemiologic Studies-Depression Scale; ETI, Early Trauma Inventory; CATS, Children’s Autonomic Thoughts Scale. * *p* < 0.05, ** *p* < 0.01.
